# The Reconstruction of Human Fingerprints From High-Resolution Computed Tomography Data: Feasibility Study and Associated Ethical Issues

**DOI:** 10.2196/38650

**Published:** 2022-11-23

**Authors:** Orestis L Katsamenis, Charles B Burson-Thomas, Philip J Basford, J Brian Pickering, Martin Browne

**Affiliations:** 1 µ-VIS X-ray Imaging Centre Faculty of Engineering and Physical Sciences University of Southampton Southampton United Kingdom; 2 Bioengineering Science Research Group Faculty of Engineering and Physical Sciences University of Southampton Southampton United Kingdom; 3 Electronics and Computer Science Faculty of Engineering and Physical Sciences University of Southampton Southampton United Kingdom

**Keywords:** microcomputed tomography, μCT, microCT, HRpQCT, fingerprints, fingerprint, ethics, ethical, image, data, biometric, information, human, hands, x-ray, CT, care

## Introduction

Volumetric imaging with modalities like magnetic resonance imaging (MRI) or x-ray computed tomography (CT) has been a valuable tool in many areas of clinical, preclinical, and basic research. Image files are data rich, often with metadata containing (or linked to) both *personal* and *sensitive* data like the name of the participant or diagnoses. When it comes to sharing these data for research, current ethics consensus and legislation require all data that can be used to link the shared data set directly or indirectly to the participant to be carefully removed [1]. This is of high importance as the deidentified data sets ensure the protection of participant confidentiality. However, in some cases, the information that can link the participant to the data is not simply accompanying the data as metadata. It *is* (part of) the data as, for example, in the case of head imaging where the data set can be used to reconstruct an image of participants’ faces at a sufficient quality that can be then used for the reidentification of the participants [2].

However, the head is not the only site from where unique biometric information can be extracted. Perhaps the most widely used biometric information for human individualization is the friction ridge pattern on the fingertips, often referred to as fingerprints [3]. As with the face in head imaging, friction ridges are also recorded as part of the imaging data when a participant’s hand is imaged. High-resolution x-ray CT imaging modalities such as high-resolution peripheral quantitative CT and microfocus CT (μCT) are used to image human hands for accessing the trabecular structure or various pathological changes (eg, osteoarthritis) with a voxel size <100 μm [4]. Here we show for the first time that the spatial resolution provided by these technologies enables retrieval of the friction ridge pattern.

## Methods

**Figure 1:**
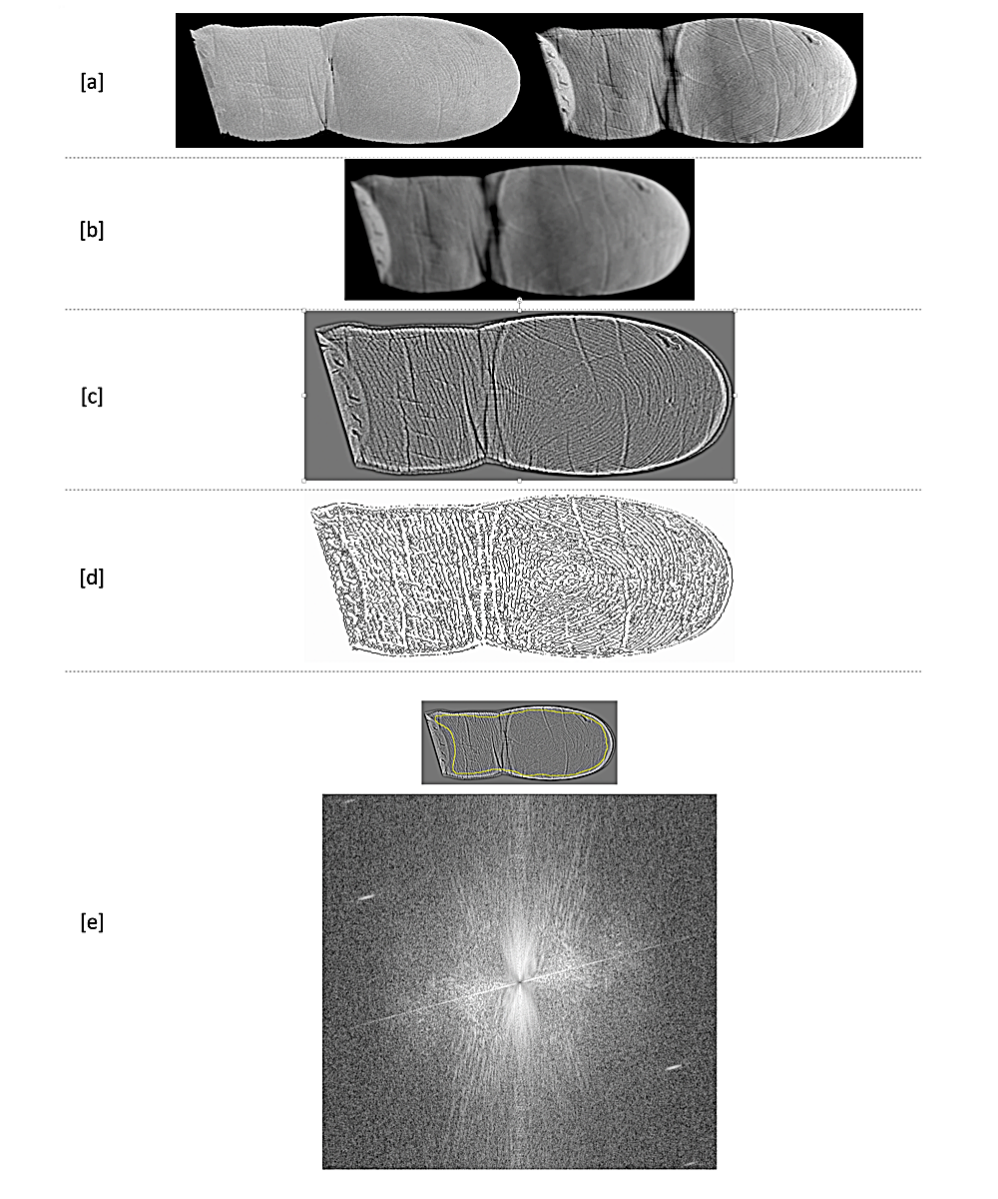
Fiji/ImageJ processing workflow for extracting the friction ridge information from the processed volume resulting from the workflow described in Multimedia Appendix 1. (A) Maximum intensity projection of the skin layer (left) and SD projection (right), (B) blurred image of the SD projection, (C) edge image resulting from the subtraction of the blurred and SD image, (D) skeletonized image of (C), (E) frequency domain Fourier image (bottom) of the selected friction ridge area shown in the insert image (inner area of outlined region) (Multimedia Appendix 2).

A fixed cadaveric human right hand from a human body donor was imaged using a μCT system designed for 3D x-ray histology [5] at the 3D x-ray histology laboratory [6] at the University of Southampton, United Kingdom. Imaging was conducted using an isotropic voxel (edge) size of 72 μm at 160 kVp, maintaining an electron beam spot size <15 μm (Table 1). Image processing was conducted using Fiji/ImageJ [7] and volumetric renderings using VG Studio Max (v2.1.4 64-bit, Volume Graphics GmbH). Extraction of the 2D net of the ridge pattern was performed in Fiji/ImageJ, and it involved a workflow to isolate the ridge pattern–containing voxels of the outer surface of the finger, that is, the epidermis, the dermis, and part of the subcutis layer, and a method to project the pattern onto a 2D plane, which is outlined in Figure 1 (see Multimedia Appendix 1 for more details).

**Table 1:** Microfocus computed tomography imaging parameters.

Scanner used	X-ray histology scanner (Custom Nikon XT H 225 ST)
Approximate total scan time (h:min:s)	0:23:37
Detector binning	1 × (2850 × 3850 dexels)
Target material	Tungsten (W)
Acceleration voltage (kVp)	160
Current (µA)	112
Power (W)	17.92
Angular projections	2001
Frames per projection	4
Exposure per frame (ms)	177
Analog gain (dB)	24
Filter material	None
Voxel size (µm)	72.00 (isotropic)

### Ethical Considerations

The study was performed in accordance with the University of Southampton’s ethics policies and ethical guidelines (ERGO/FEPS/67396). The sample was obtained by cadaveric donors who have given consent for their bodies to be used for scientific research and imaging.

## Results

**Figure 2:**
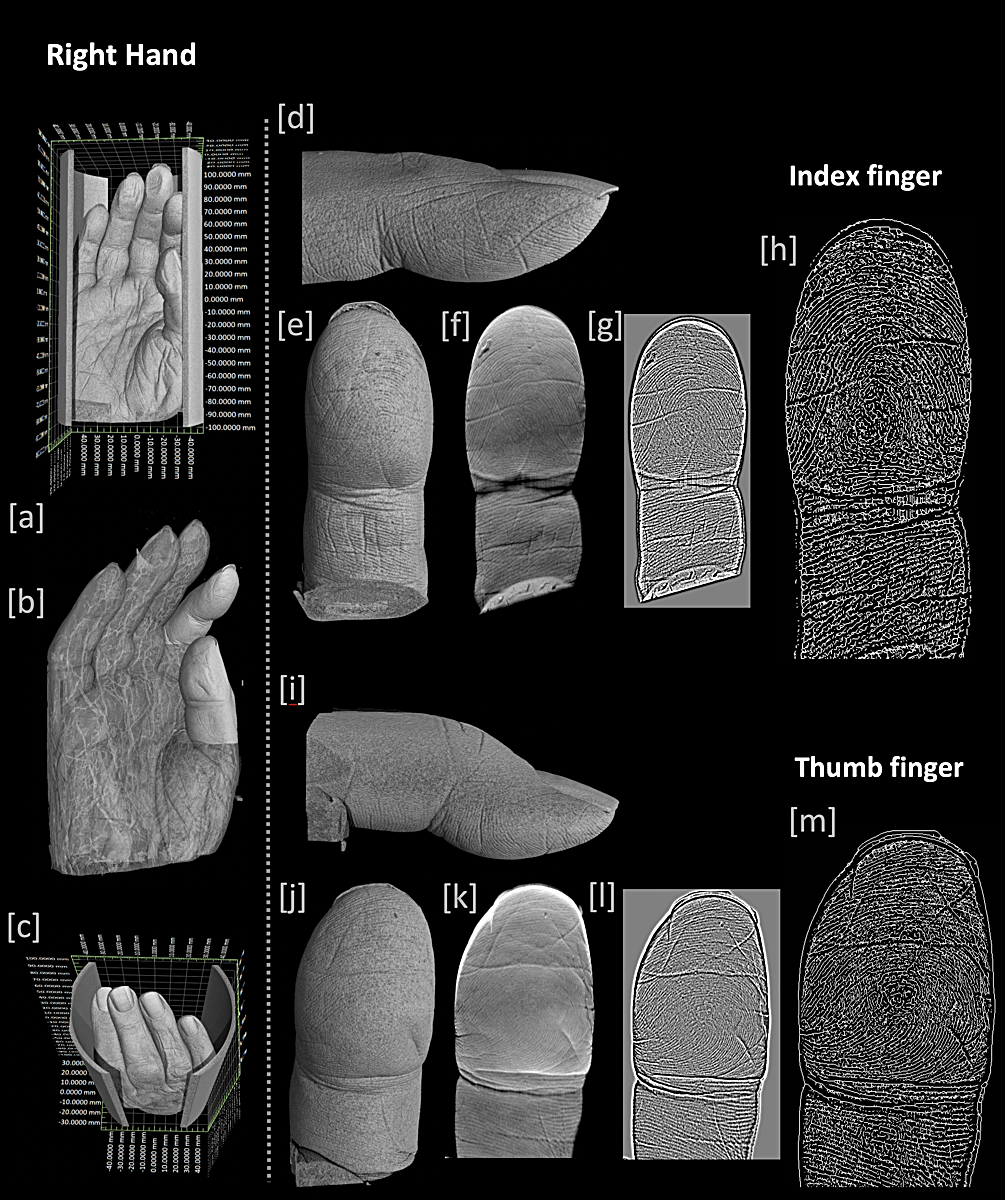
Cadaveric human right hand imaged for the needs of the Anatomically Precise Revolutionary Implant for Bone Conserving Osteoarthritis Treatment (APRICOT) project using a high-resolution x-ray microfocus computed tomography system at the x-ray histology laboratory, University of Southampton. Left pane (A-C): volume renderings of the hand depicting the positioning and the segmentation of the index and thumb fingers for further analysis. Right pane: (D,I) top and (E,J) bottom view of volumetric rendering of the segmented finger volumes. (F,K) SD projection across the segmented volume after the removal of all information deeper than 10 voxels from the surface (skin). (G,L) Edge image of (F,K) and (H,M) skeleton of the binarized (G,L) images (Multimedia Appendix 3).

Following the workflow described above and in Multimedia Appendix 1, one is left with a single volume per finger that contains only the volumetric gray-level information of the friction ridges, which can now be projected onto a single 2D plane so that the net of the friction ridge pattern can be recorded and further processed (Figures 1 and 2). Two examples of further processing are shown in Figure 1D and E, where a skeleton (1D) of the Fourier (frequency domain) image (1E) of the edge image is presented.

## Discussion

μCT imaging was sufficient to resolve the friction ridges of the fingertips. In silico reconstructed fingerprints of the thumb and the index fingers using the whole hand μCT data set are shown in Figure 2. With fingerprint-based identification being widely used outside forensics in a range of applications such as accessing personal accounts and devices, the risk of reidentification, impersonation, and password compromisation should be considered and discussed in an ethics application. Regarding the latter point, imaging scientists should be made aware that processing of such data might potentially have implications on data protection obligations; since biometric data are used for identification (eg, authentication), they become *special category personal data* (General Data Protection Regulation, article 9-1) [8]. Our study complements previous imaging studies that assessed the ethical implication associated with the risk of reidentification of the participants using MRI data [2] and highlights the risks of modern high-resolution imaging modalities, such as x-ray μCT, accidentally producing identifiable information. We argue that data sets containing high-resolution CT images of the human hands should be considered “sensitive” and thus handled and shared with appropriate care, as data subjects and imaging scientists may not be able to identify a priori all implications of further processing [9]. Relevant bodies (eg, institutional ethics committees or data protection committees) should consider this aspect of high-resolution CTs when reviewing research or clinical imaging protocols and make their recommendations according to the currently applicable law and local code of practice.

## Appendix

Multimedia Appendix 1 Supplementary information.

Multimedia Appendix 2 Supplementary Figure 1.

Multimedia Appendix 3 Supplementary Figure 2.

## Abbreviations

CT: computed tomography
MRI: magnetic resonance imaging
μCT: microfocus computed tomography

## Data Availability

Data generated during the study are available from the corresponding author by request.
