# Supplementary material for: The Reconstruction of Human Fingerprints From High-Resolution Computed Tomography Data: Feasibility Study and Associated Ethical Issues
Source: J Med Internet Res. 2022 Nov 23;24(11):e38650. doi: 10.2196/38650 (PMC9730206; doi:10.2196/38650)
Supplement: Multimedia Appendix 1 [file jmir_v24i11e38650_app1.docx]

## **Supplementary information – The Reconstruction of Human Fingerprints From High-Resolution Computed Tomography Data: Feasibility Study and Associated Ethical Issues**

## Orestis L. Katsamenis^1*^, Charles B. Burson-Thomas^2^, Philip J. Basford^1^, J. Brian Pickering^3^, Martin Browne^2^

**^1^** μ-VIS X-ray Imaging Centre, Faculty of Engineering and Physical Sciences, University of Southampton, Southampton, United Kingdom; **^2^** Bioengineering Research Group, Faculty of Engineering and Physical Sciences, University of Southampton, Southampton, United Kingdom; **^3^** IT Innovation, Electronics and Computer Science, Faculty of Engineering and Physical Sciences, University of Southampton, Southampton, United Kingdom

***Specimen and Data acquisition***

A cadaveric human right hand imaged for the needs of the “*Anatomically Precise Revolutionary Implant for bone Conserving Osteoarthritis Treatment (APRICOT)*”^[[1]](#footnote-1)^ project using a high-resolution X-ray microfocus CT system at the 3D X-ray histology laboratory^[[2]](#footnote-2)^ at the University of Southampton, UK. The study was performed in accordance with the University of Southampton's (Southampton, UK) ethics policies and ethical guidelines (ERGO/FEPS/67396). Sample was obtained by cadaveric donors who have given consent for their body to be used for scientific research and imaging.

Imaging was conducted using a micro-CT scanner designed for 3D X-ray histology [5] at isotropic voxel (edge) size of 72 μm using a 225kVp source driven at 160 kVp maintaining an electron-beam spot size spot-size < 15 μm (Figure 1). The acquisition parameters shown in table 1. Image processing was conducted using Fiji/ImageJ [7] and volumetric renderings using VG Studio Max (v2.1.4 64 bit, Volume Graphics GmbH, Germany).


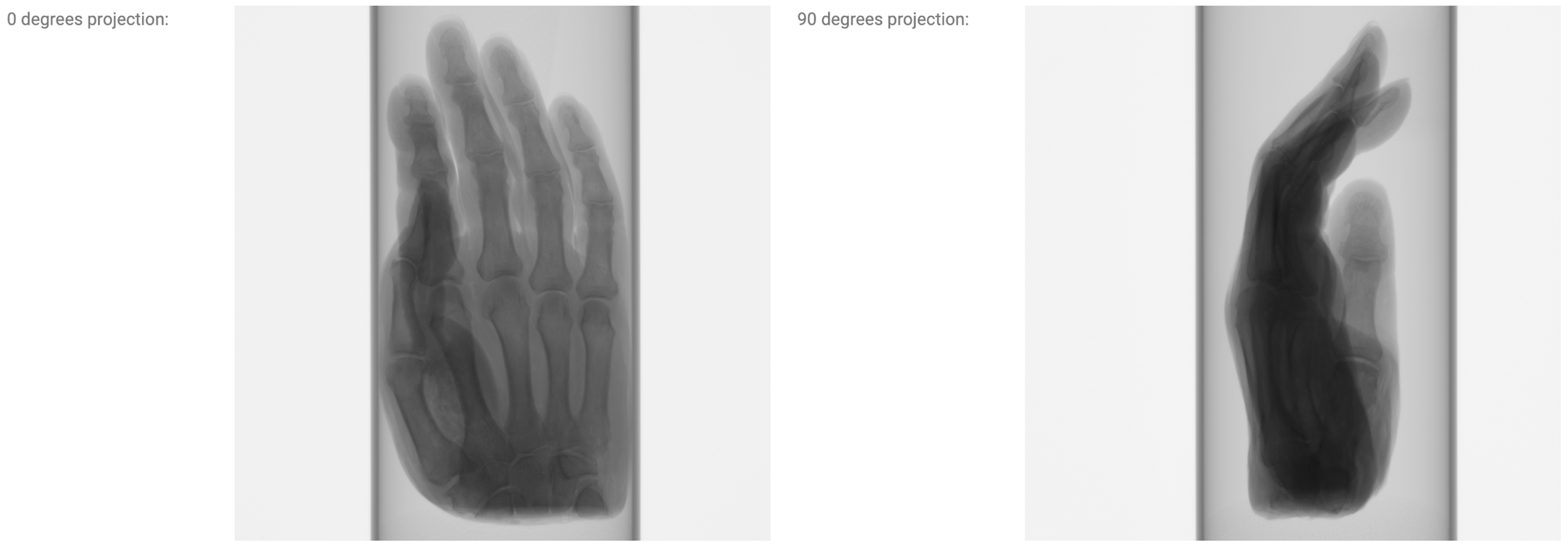


**Figure 1**: 0 and 90 degrees radiographs of the sample within the Perspex tube used for mounting the specimen into the X-ray μCT scanner.

| Table 1: μCT imaging parameters | |
| --- | --- |
| Scanner used | **X-ray Histology Scanner (Custom Nikon XTH225XT)** |
| Total scan time (approx) | 0:23:37 |
| Detector Binning | 1x (2850 x 2850 dexels) |
| Target material | Tungsten (W) |
| Acceleration voltage | 160 kVp |
| Current | 112 µA |
| Power | 17.92 W |
| Angular projections | 2001 |
| Frames per projection | 4 |
| Exposure per frame | 177 ms |
| Analog Gain | 24 dB |
| Filter material | None |
| Voxel size | 72.00 µm (isotropic) |

***Volume data post processing and friction ridge pattern extraction***

Following μCT imaging the data were reconstructed to 32-bit raw volume files using Nikon’s CT reconstruction software (CTPro, version V6.7.7885.27111; Nikon X-Tek Systems, Tring, UK) using conventional filtered back projection. The reconstructed 32-bit raw volumes were imported into Fiji/ImageJ (v1.51n), where a 3D median filter (1×1×1 kernel) was applied, followed by a 2D un-sharp mask (Gaussian blur factor = 2 pixels, applied on each reconstructed slice of the CT stack). Grey levels were linearly windowed to [-50, +100] and saved as 16-bit volumes. The resultant volume was further reduced to 8-bit using a 16 to 8-bit window of [20000 41000] to generate a more manageable volume size. The final c. 4 GB volume was then imported into VG Studio Max, which was used to [a] segment and export the index and thumb finger sub-volumes and [b] render all the volume images shown in the main text; i.e. sub-panels [a – e, I ,j ].

***Extraction of the 2D net of the ridge pattern***

Extraction of the 2D net of the ridge pattern was performed in Fiji/ImageJ and it involved a workflow to isolate the ridge pattern containing voxels and the projection of this information onto the 2D plane. The workflow is described below and in Figure 2

1. volume image is loaded
2. volume is sequentially rotated and resliced so that the orthogonal planes run parallel and normal to the length of the finger as shown in Figure 2a
3. A (volume) mask is then generated (0=air; 1=tissue) using threshold (Figure 2b)
4. The mask was eroded and dilated (5x voxels for index, 7x voxels for thumb) generating two new (volume) masks as per in Figure 2c
5. The eroded and dilated mask were then combined (XOR) to generate a segmentation mask which includes information only from the skin area (Figure 2c)
6. The resulting mask was then multiplied with the 8-bit volume and resliced (bottom to top) up to the nailbed (Figure 2e), resulting into the resliced segmented 8-bit volume of air + skin information (Figure 2f). This volume is oriented such as the XY-plane runs parallel to the nailbed and in line to what would be the fingerprint scanner or fingerprint card orientation.


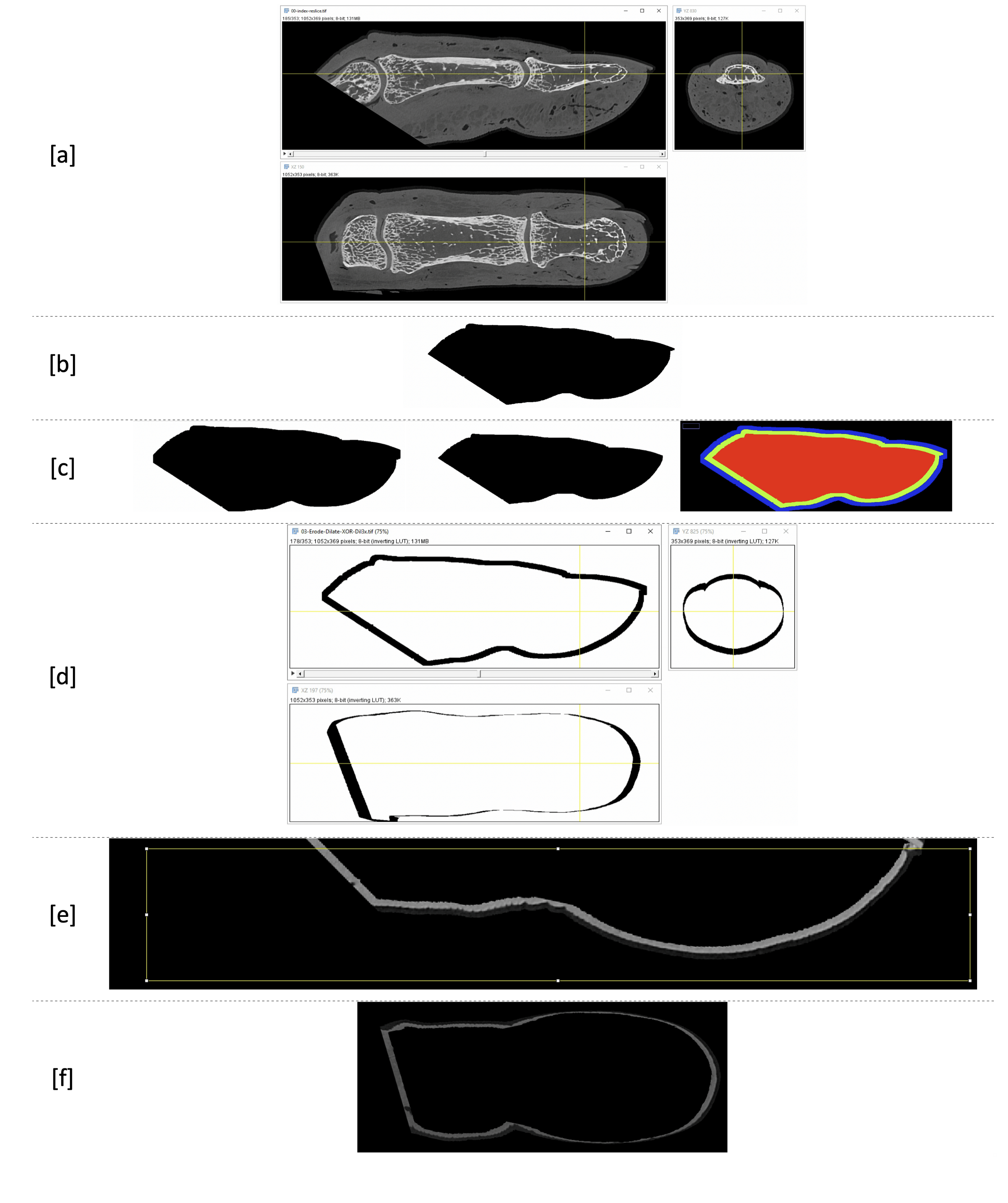


**Figure 2**: Fiji/ImageJ processing workflow for segmenting the friction ridge containing voxels using a single finger sub-volume. [a] Orthogonal planes across the aligned volume; [b] threshold-based (volume) mask; [c] dilated mask (left), eroded (middle) mask and composite image showing the eroded (red) and the dilated (blue) masks on the initial mask (green); [d] Orthogonal planes across the resultant XOR mask of the eroded (red) and the dilated (blue); [e] representative slice of the segmented (skin only) volume and the selected region of interest across which the volume is resliced (bottom to top); [f] single slice from final 8-bit volume of air + skin information. This volume is oriented such as the XY-plane runs parallel to the nailbed.

1. https://www.apricot-project.eu [↑](#footnote-ref-1)
2. https://www.xrayhistology.org [↑](#footnote-ref-2)
