# Supplementary figures and images for: The Reconstruction of Human Fingerprints From High-Resolution Computed Tomography Data: Feasibility Study and Associated Ethical Issues
Source: J Med Internet Res. 2022 Nov 23;24(11):e38650. doi: 10.2196/38650 (PMC9730206; doi:10.2196/38650)

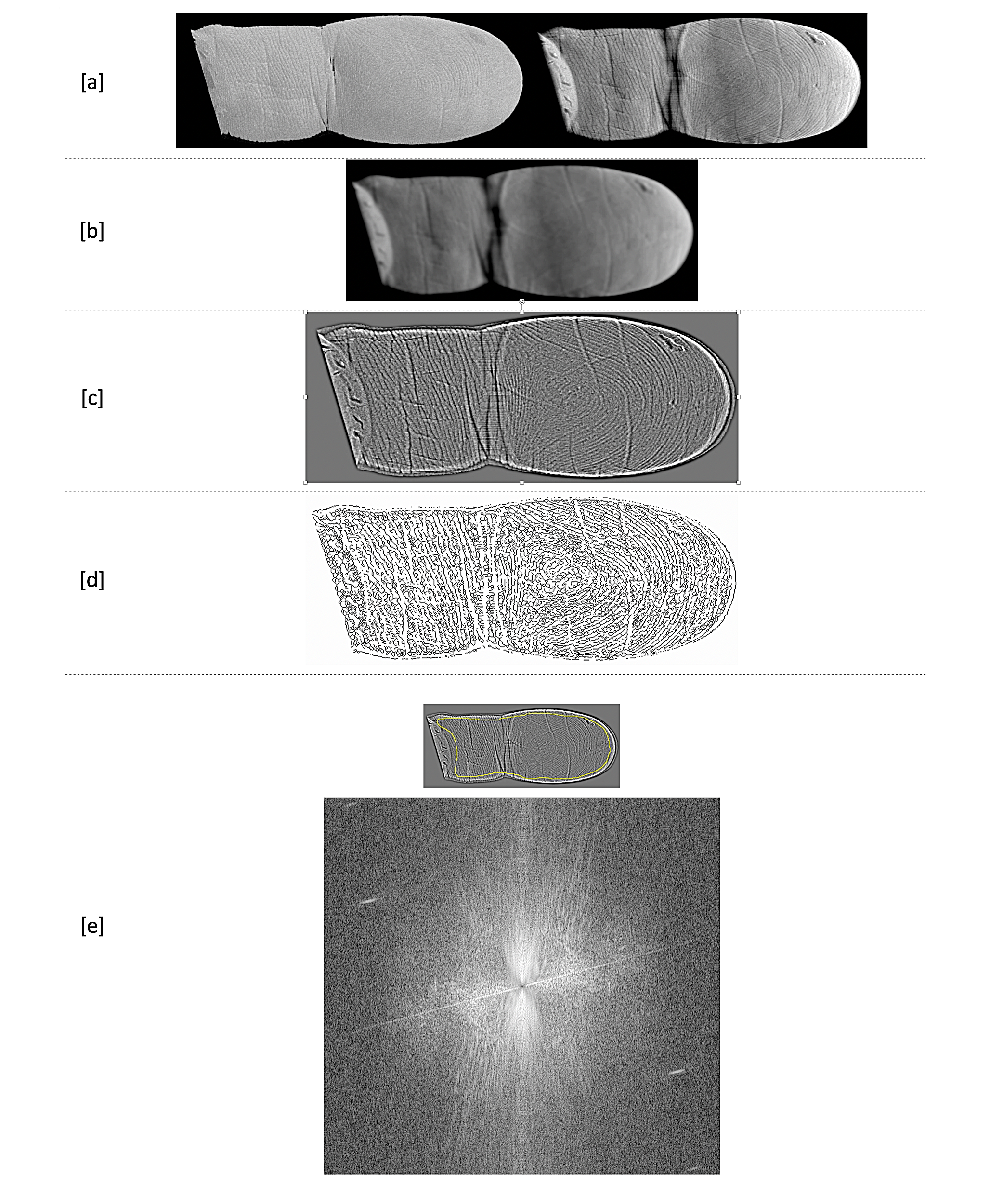

Supplement: Multimedia Appendix 2 [file jmir_v24i11e38650_app2.png]

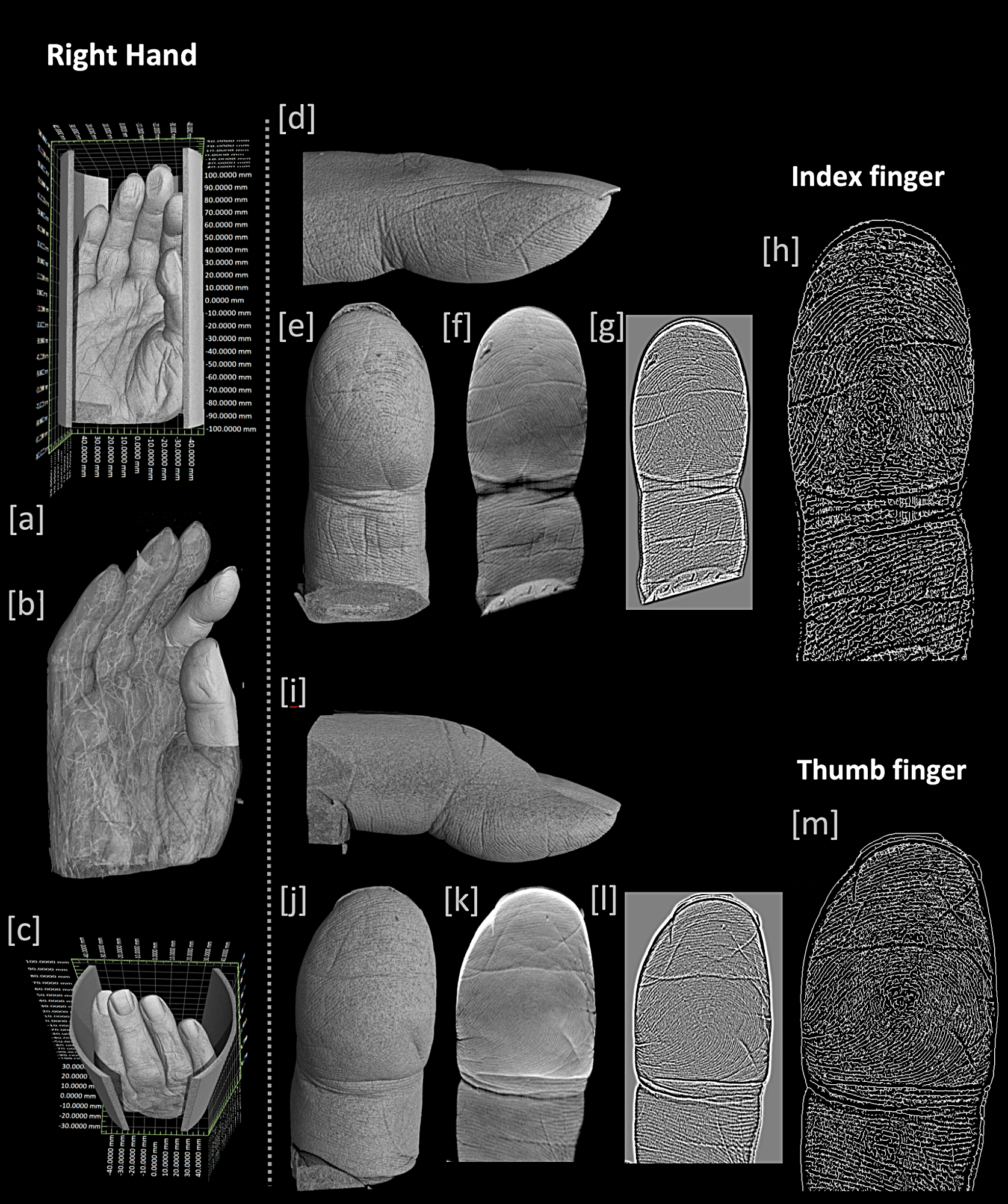

Supplement: Multimedia Appendix 3 [file jmir_v24i11e38650_app3.png]
